# Individual Tree Crown Delineation Using Multispectral LiDAR Data

**DOI:** 10.3390/s19245421

**Published:** 2019-12-09

**Authors:** Faizaan Naveed, Baoxin Hu, Jianguo Wang, G. Brent Hall

**Affiliations:** 1 Department of Earth and Space Science and Engineering, York University, Keele Street, Toronto 4700, ON M3J 1P3, Canada; fazanham@my.yorku.ca (F.N.); jgwang@yorku.ca (J.W.); 2 Education and Research, Esri Canada, 900-12 Concorde Pl, Toronto 4700, ON M3C 3R8, Canada; bhall@esri.ca

**Keywords:** multispectral LiDAR, individual tree crown delineation, seeded region growing, multi-scale analysis, neutrosophic logic

## Abstract

In this study, multispectral Light Detection and Ranging (LiDAR) data were utilized to improve delineation of individual tree crowns (ITC) as an important step in individual tree analysis. A framework to integrate spectral and height information for ITC delineation was proposed, and the multi-scale algorithm for treetop detection developed in one of our previous studies was improved. In addition, an advanced region-based segmentation method that used detected treetops as seeds was proposed for segmentation of individual crowns based on their spectral, contextual, and height information. The proposed methods were validated with data acquired using Teledyne Optech’s Titan LiDAR sensor. The sensor was operated at three wavelengths (1550 nm, 1064 nm, and 532 nm) within a study area located in the city of Toronto, ON, Canada. The proposed method achieved 80% accuracy, compared with manual delineation of crowns, considering both matched and partially matched crowns, which was 12% higher than that obtained by the earlier marker-controlled watershed (MCW) segmentation technique. Furthermore, the results showed that the integration of spectral and height information improved ITC delineation using either the proposed framework or MCW segmentation, compared with using either spectral or height information individually.

## 1. Introduction

Individual tree analysis serves as a foundational process in various fields, such as forestry, environmental protection, and power line management. Analyses pertaining to individual tree crowns (ITCs) critically rely on accurate delineation of crowns, as delineated ITCs are commonly used to estimate the sizes, ages, and heights of tree crowns [1,2,3], perform tree species classification [4,5], and monitor tree growth [6]. For the past several decades, many studies on ITC delineation have been conducted using high spatial resolution imagery. Some of the most popular delineation methods include edge detection [7,8], region growing [3,9], and watershed segmentation [10,11]. Even though these methods have achieved satisfactory results, incomplete crown edges are often detected due to the variation in illumination between individual crowns. Furthermore, high commission errors are commonly observed in dense tree stands due to the minimal variation in reflectance between neighbouring crowns. Due to the increasing accessibility of Light Detection and Ranging (LiDAR) data, utilization of these data in ITC delineation to identify the structural differences, canopy boundaries, and spaces among crowns is gaining more attention. LiDAR point clouds are often rasterized to generate canopy height models (CHMs) in which the local maxima and neighbouring pixels with lower elevations represent treetops and canopies, respectively. Even though detailed crown profiles in LiDAR data allow for accurate ITC delineation in open forests (similar to passive optical imagery), there remain problems in deciduous or mixed forest stands [12], namely tree crowns tend to have varied sizes and overlaps, forming tree clusters without discernible structural parameters for ITC delineation. 

Studies have indicated that the structural information from LiDAR data is useful for identification of treetops, but spectral information from optical imagery is better for discerning the boundaries between adjacent crowns [13,14]. As a result, several studies have used the structural and spectral differences among tree crowns in LiDAR data and passive optical imagery, respectively, to improve ITC delineation. In most of the methods that these studies have applied, a CHM derived from LiDAR data is utilized to identify treetops and the reflectance from optical imagery is used for delineation of crown boundaries. Delineation is usually performed with a seeded region growing (SRG) or marker-controlled watershed (MCW) segmentation method, in which the detected treetops are used as seeds or markers, respectively [13,14]. A limited number of studies have examined the benefits of integrating LiDAR data with optical imagery at the crown delineation phase [14]. Recently, Lee et al. [15] proposed an integration strategy that applies a graph cut approach to generate graph weights based on both optical imagery and LiDAR data, reporting accuracy (i.e., the ratio of extracted trees to matched trees) of around 50% for coniferous stands. Lee et al. [15] also stated that the mis-match in spatial resolution between the LiDAR data and optical imagery and issues regarding co-registration made it difficult to analyse the benefits of integrating spectral and structural information for ITC delineation.

As illustrated by Lee et al. [15], successful integration of LiDAR data and optical imagery critically relies on the spatial resolution, coverage, and minimal relief of the optical imagery. These requirements are difficult to satisfy, particularly if the datasets being integrated are collected from different missions at different times and with different configurations (e.g., flying height, view angle, scan direction). Even after these differences are rectified, problems still exist due to shadows and registration errors [15]. Current advances in multispectral LiDAR technologies provide a good opportunity to improve ITC delineation using spectral and structural information without encountering the frequent problems associated with the integration of datasets from different missions and configurations.

Multispectral LiDAR instruments are capable of providing 3D coordinates of surface objects and their reflected intensities at several electromagnetic wavelengths. For example, some multispectral LiDAR instruments collect three different point clouds at three wavelengths (1550, 1064 and 532 nm). Since the three point clouds (and their 3D coordinates and intensities) are collected simultaneously and with similar configurations, minimal processing is required to co-register them. A few studies have used multispectral LiDAR data for land cover classification [16,17,18]. Hence, the goal of this research was to exploit the capability of multispectral LiDAR data to improve ITC delineation by fully and effectively utilizing combined structural and spectral information about tree canopies. In most ITC methods, treetops are detected first and then segmentation methods, such as SRG and MCW, are used to delineate crown boundaries. This study sought to improve treetop detection and crown delineation by designing a framework to integrate structural and spectral information about crowns. It is worth mentioning that while preparing this manuscript, it was brought to our attention that Dai et al. [19] investigated the application of multispectral LiDAR data to improve ITC delineation. Adetailed comparison between this study and [19] will be provided in the discussion section. 

Most methods for detecting treetops [20,21] rely on filtering techniques to identify the local maxima (e.g., treetops) in a scene. However, as crown sizes vary, such methods yield high commission errors due to false detection of treetops. As a result, an increasing number of researchers prefer multi-scale approaches [11,12]. The method proposed by Jing et al. [11] and Hu et al. [12] is advantageous compared to most existing methods because instead of detecting whether individual pixels are treetops, it enables detection of the largest horizontal cross-sections of tree crowns, which are not sensitive to noise in the data. However, Jing et al. [11] and Hu et al. [12] only proposed a simplified approach for merging treetops detected at different scales based on a measure of region circularity. Thus, their method was sensitive to the selected range of scales (reflecting the dominant crown sizes in the scene) that was difficult to determine [12]. To overcome this issue, this study proposes an advanced merging strategy to integrate effectively identified treetops at different scales.

Regarding integration of the spectral and structural features of crowns for delineating crown boundaries, existing segmentation methods with region-based approaches are advantageous due to their ability to combine information from different data sources. Using a pre-defined merging criterion, region-based methods iteratively merge single pixels with their adjacent segments. The merging criterion provides a mechanism to incorporate features related to the spectral, contextual, and structural characteristics of tree crowns that are derived from different data sources.

Even though various studies have proposed ways to improve region-based ITC delineation methods, most have focused on individual data sources [14,22]. Of the few studies that combine spectral and structural information for ITC delineation, most simply append the CHM to optical imagery as an extra band [13,14]. Such approaches fail to exploit fully crown morphology by treating the CHM as another spectral band. In this study, height information in the CHM was used to constrain the growth of segments, accounting for the shape of crowns. In addition, most existing merging criteria are based on the similarity/difference between a given segment and its neighbouring pixels [23]. The contextual information associated with individual pixels to be merged is rarely considered, and during the merging process, a pixel within a homogeneous area (i.e., tree crown or background) is commonly treated as existing within the boundary between tree crowns or between a tree crown and background. In this study, contextual information was considered based on a neutrosophic logic approach that was created by Smarandache [24]. In neutrosophic logic, a logical variable is associated with three components, the degrees of truth, indeterminacy, and falsehood. It can be appliedto uncertainty and ambiguity in data and mathematical models. It was first used by Shan et al. [25] to segment tumors in noisy ultrasound images. In addition, to define the similarity measure, or the degree of truth in neutrosophic logic [24,25], a degree of indeterminacy was employed. This degree of indeterminacy was measured as the variance within a small area surrounding the pixel of interest, which determines how the pixel could be considered during the merging process. A large degree of indeterminacy means that the pixel is situated within a noisy area and the features of the area, rather than the features of the pixel, should be considered. In the context of ITC delineation, a small degree of indeterminacy implies that the area around the pixel is homogeneous and, perhaps, contains a single tree crown, while a large degree of indeterminacy implies that the area is more heterogeneous and may be at the boundary of a crown. We investigated neutrosophic logic as a way to improve ITC delineation in a previous study, and the preliminary results indicated that it was promising [26]. In this study, the merging criteria based on neutrosophic logic were further developed to provide a framework to include contextual information and integrate features from different data sources that may have different uncertainties. 

The ITC delineation method was tested on a set of multispectral Titan data obtained within the study area, which was located in the city of Toronto, ON, Canada (described in detail in Section 2). The results were compared with manually delineated tree crowns and with the results obtained by MCW methods. In addition, two variants of the developed method were applied to LiDAR intensities and CHM individually to test the effectiveness of a strategy that combined LiDAR intensities and CHM.

## 2. Materials and Methods

### 2.1. Study Area and Data Used

**Figure 1:**
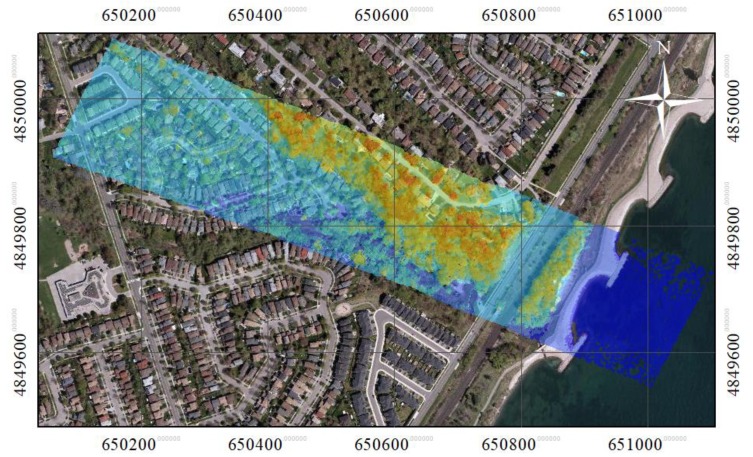
The LiDAR point cloud in the study area overlaid on a true-colour aerial image (Credits: Esri, HERE, Garmin, © OpenStreetMap contributors, and GIS user community. Source: Esri, DigitalGlobe, GeoEye, EarthStar Geographics, CNES/Airbus DS, USDA, USGS, AeroGRID, IGN, and the GIS User).

The dataset used in this study was acquired in September 2014 from a Titan multispectral sensor (Teledyne Optech, Concord, ON, Canada), operating at three wavelengths: 1550 nm (Channel 1), 1064 nm (Channel 2), and 532 nm (Channel 3), and mounted on an aircraft. The three channels were collected at different viewing angles: 1550 nm at 3.5° forward-looking, 1064 nm at 0° nadir-looking, and 532 nm at 7° forward-looking. Three separate data clouds were generated, and their point densities were slightly different but around 45 pts/m^2^ for individual data clouds. The three point clouds in the Titan dataset were co-registered by generating a reference grid. The reference grid was formed using channel 1 (1550 nm). Even though channel 1 was used to generate the reference grid, it was observed that the selection of a different channel did not affect the spatial resolution of the grid or the quality of interpolation. The datasets were collected in a study area located in the south-eastern region of Toronto, ON, Canada. The point cloud in channel 1 is overlaid with an aerial image in Figure 1. As shown in the figure, the scene consists of isolated trees located in urban zones (to the northwest) and dense tree clusters in woodlots (to the southeast). The tree crowns are dominantly deciduous. During the data acquisition, the deciduous trees were with leaves on and mainly green. 

**Figure 2:**
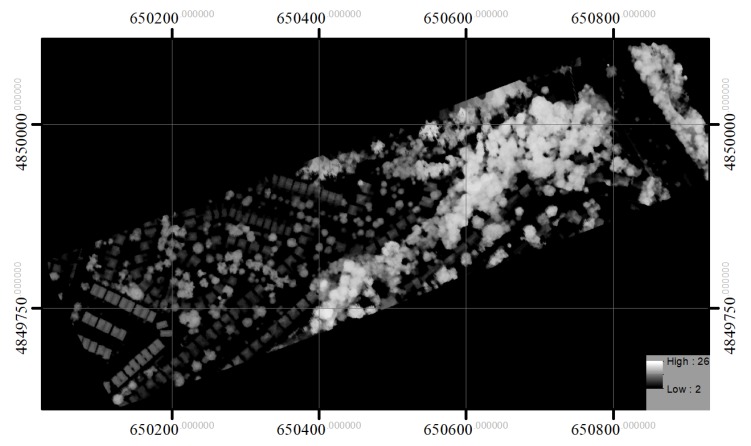
The CHM of the study area.

**Figure 3:**
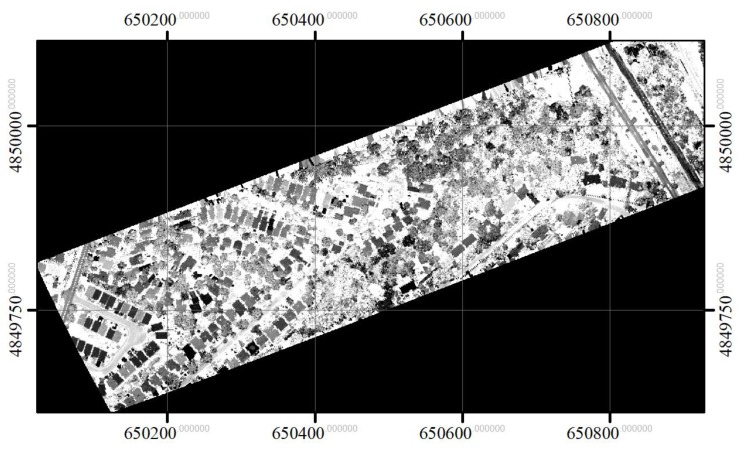
Range normalized intensity image in channel 1 (1550 nm).

**Figure 4:**
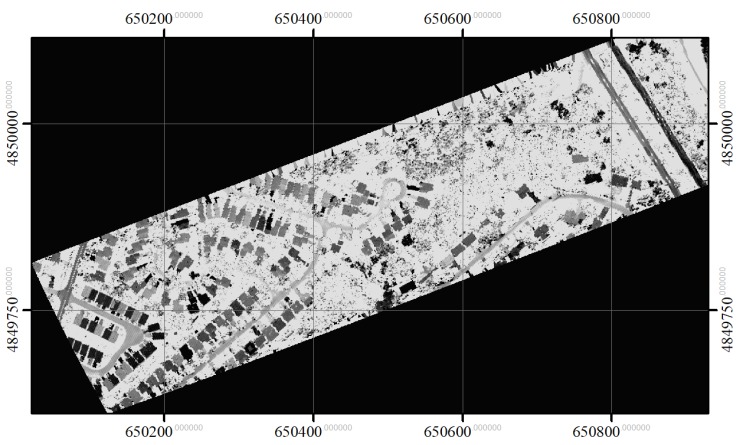
Range normalized intensity image in channel 2 (1064 nm).

**Figure 5:**
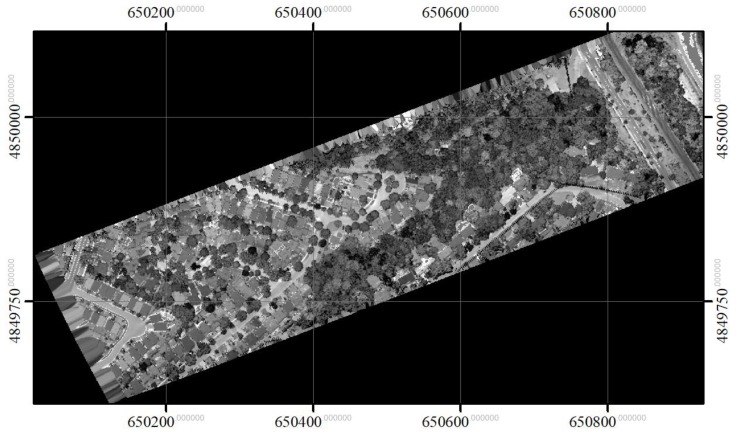
Range normalized intensity image in channel 3 (532 nm).

The intensities recorded for the three channels were first normalized with respect to range [27]. A digital surface model (DSM) and intensity image for each channel were generated by rasterizing the first returns. The spatial resolution of the raster images was 0.25 × 0.25 m based on the average distance between pulses [21]. To generate the DSM and intensity image, the maximum height value and its corresponding intensities were used for each cell with first-return LiDAR points, and any empty cell was filled by interpolating the values of non-zero neighbour cells using inverse distance weighted interpolation [28,29]. The CHM image was created based on the difference between the DSM and a digital elevation model (DEM), which was generated using a progressive TIN densification algorithm (Axelsson [30]). The CHM was then smoothed with a 3 × 3 Gaussian low-pass filter to eliminate noise, as done by Hyyppä et al. [28] and Morsdorf et al. [31]. The three CHMs generated for the three channels were similar, and, as a result, the CHM for channel 1 (1550 nm) was used to obtain height information about individual crowns. The three intensity images were used to obtain spectral information about the tree crowns. Figure 2, Figure 3, Figure 4, and Figure 5 show the generated CHM and three intensities images, respectively.

In the final pre-processing stage, non-tree pixels were masked using both the CHM and normalized intensity images. A normalized differenced vegetation index (NDVI) was generated by using the normalized intensity for channel 3 (550 nm) and channel 1 (1064 nm) to identify vegetation pixels. The CHM was then used to filter out non-tree pixels. A non-tree pixel was defined as a pixel for which the NDVI value was below a threshold of 0.25 and the elevation in the CHM was below a threshold of 4 m. 

### 2.2. Methodology

#### 2.2.1. Individual Treetop Detection

An existing multi-scale treetop detection method was improved to mitigate false detection of treetops. In a previous study, Hu et al. [12] developed a multi-scale treetop detection method to account for different crown sizes in a scene. Instead of detecting single points for treetops, similar to most ITC methods, the multi-scale approach detected a large horizontal cross-section of individual trees, and a 3D cross-section of a crown, obtained from a near-nadir perspective, was visualized as half of an ellipsoid [12]. Any single cross-section of the ellipsoid was viewed as a disk with a certain diameter, which could be used to identify the width of the crown at that elevation. A morphological opening process with a disk structuring element (SE) was proposed to remove objects (i.e., upper crown branches) smaller than the specified kernel. The maxima in an opened image represent a cross-section of the tree crown at the corresponding scale. Applying the opening process to a CHM image with a series of disk SEs of different sizes would generate a series of cross-sections of individual tree crowns. These cross-sections represent treetops at different scales. The detected treetops were then merged to generate the final treetop image. Hu et al. [12] employed a simple criterion based on the circularity of the detected cross-sections to find the maximum cross-section (across multiple scales) that was most circular. In this study, a new merging strategy was developed.

Based on the method described by Hu et al. [12], we first identified the range of scales corresponding to the predominant crown sizes in the scene. A series of disk SEs with diameters (i.e., scales) ranging from 3 to 73 pixels (in increments of 2 pixels) were used to generate morphologically opened images. The mean values of the morphologically opened images at two adjacent disk SE diameters were differenced (the image with the larger SE diameter was used as the minuend). The numerically big difference in the mean between these images indicated that there were a relatively large number of tree crowns whose sizes were equal to the disk with the diameter of the opened image used as the minuend. The dominant crown sizes were then determined based on the local minima of the plot of the mean versus the disk SE diameters. Since several sizes of SEs were employed for treetop detection, the method is referred to as multi-scale analysis. It is worth mentioning that with the morphological analysis, the dominant sizes of objects within the scene could be determined by the local minima of the plot of the mean versus the disk SE diameters. However, it is hard to determine the range of scale sizes for tree crowns. A small-scale size might represent the size of big branches and a large-scale size might correspond to cluster of trees. The range of scale sizes that corresponded to tree crowns could be determined based on a prior knowledge on the sizes of small and large crowns. In this study, to minimize the effect of the selected range of the crowns size on the results, we implemented a statistical test in the processing to merge treetops detected at different scales.

**Figure 6:**
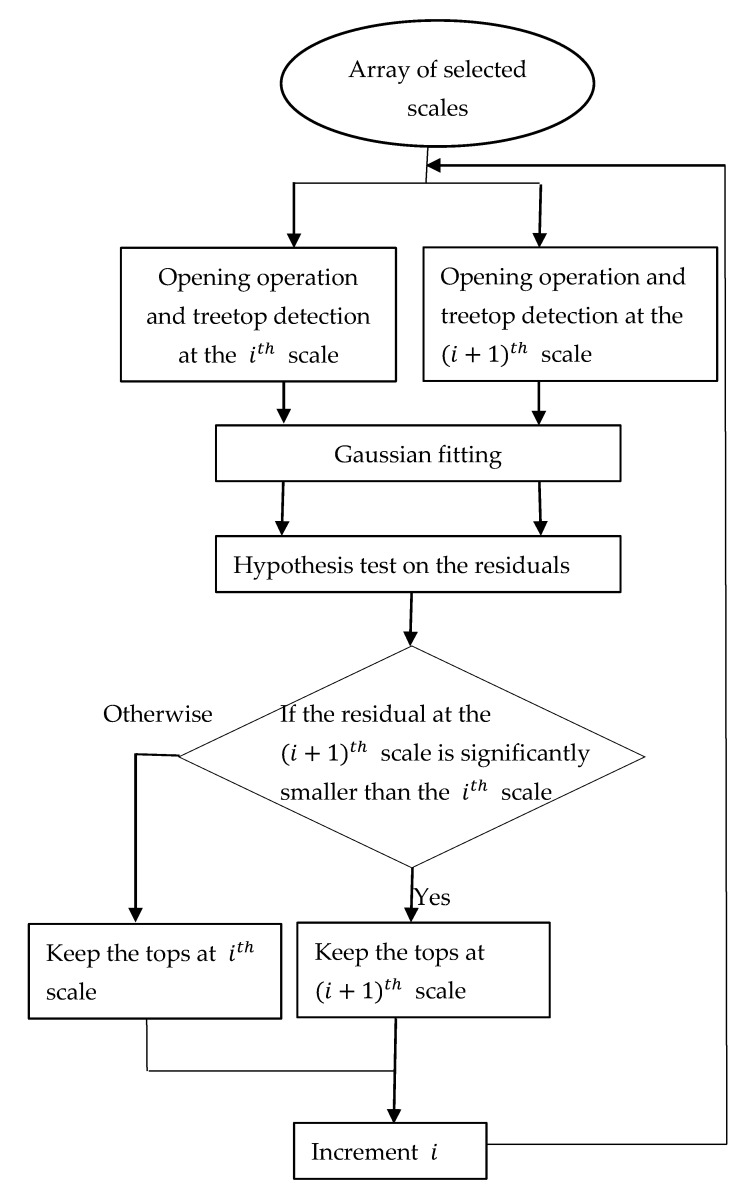
The flowchart for the multiscale treetop detection.

The key aspect of multi-scale treetop detection is integration of tree crowns detected at two adjacent scales (i.e., disk SE diameters). There were usually two potential scenarios: (1) the treetops identified at both scales were concentric (or approximately concentric) or (2) the treetops identified at the upper scale covered (or partially covered) multiple treetops at the lower scale. In the first scenario, which treetops are used is not critical. In this study, treetops at the upper scale were used and thus the cross-section used to represent a treetop was larger. However, in the second scenario, the treetops detected at the lower scale could represent the crowns and thus they should not be merged or be represented by a single treetop at the upper scale. On the other hand, if the treetops at the lower scale include the tops of big branches in a single tree crown, they need to be merged and the treetop at the upper scale is correct. An iterative process of multi-scale treetop detection was implemented. As shown in Figure 6, during each iteration, the CHM was morphologically opened at the lower ($i^{th}$) and next highest (($i+1{)}^{th}$) scales. Local maxima were detected to identify the cross-sections of tree crowns at both scales. To determine which scale represented the largest cross-section of a given tree crown, two analyses were carried out based on the CHM and intensity data. 

The first analysis was based on the CHM. It was assumed that the uppermost part of a tree crown could be described by a Gaussian function [12], and, as a result, the Gaussian function shown in Equation (1) was fitted for each identified treetop (cross-section):

(1) $$G\left(x,y\right)=Ae^{-\left(\frac{{\left(x-u_{x}\right)}^{2}}{2\sigma_{x}^{2}}+\frac{{\left(y-u_{y}\right)}^{2}}{2\sigma_{y}^{2}}\right)},$$

where $\mu_{x}$ and $\mu_{y}$ are the means of the Gaussian function and represent the center (i.e., the real treetop) of a crown; $\sigma_{x}^{2}\;$ and $\sigma_{y}^{2}$ are the variances and reflect the shape of the tree crown; A is a coefficient; and $\mu_{x},\;\mu_{y},\;\sigma_{x}$, $\sigma_{y}\;$ and A were determined by minimizing the residual defined in Equation (2):

(2) $$\varepsilon^{2}=\frac{1}{n}\;\sum_{j}^{n}{\left[CHM\left(x_{j},y_{j}\right)-G\left(x_{j},y_{j}\right)\right]}^{2},$$

where $\varepsilon^{2}$ was the residual; n was the number of pixels within the detected treetop; and $CHM\left(x_{j},y_{j}\right)$ and $G(x_{j},y_{j}$) were the observed and modelled canopy heights at point $\left(x_{j},y_{j}\right)$. Then, a statistical test was performed to determine whether the residual obtained at the upper ($i+1{)}^{th}$ scale was significantly higher than that at the lower $i^{th}$ scale at a significance level of 0.05. The statistic employed for this test is shown below:

(3) $$F=\frac{\varepsilon_{i+1}^{2}}{\sum_{k=1}^{m}\varepsilon_{k,i}^{2}},$$

where F is the test statistic, and m is the number of treetops that were detected at the $i^{th}$ scale and overlapped with the treetop detected at the ($i+1{)}^{th}$ scale.

The second analysis was based on intensity data. It was assumed that variations in intensities within a tree crown were small. For each detected treetop, variances in intensities were calculated and a statistical test was implemented to determine if the variance at the upper ($i+1{)}^{th}$ scale was significantly higher than that at the lower $i^{th}$ scale at a significance level of 0.05. The F statistic employed for this analysis is shown below:

(4) $$F=\frac{\sum_{l=1}^{3}s_{l,i+1}^{2}}{\sum_{k=1}^{m}\sum_{l=1}^{3}s_{l,k,i}^{2}},$$

where l  is the index for spectral channels; $s_{l,i+1}^{2}$ is the variance calculated for the treetop detected at the upper scale ($i+1{)}^{th}$ for channel l; and $s_{l,k,i}^{2}$ is the variance calculated for treetop k detected at the lower scale $i^{th}$ for channel l. If any of the above-mentioned F-tests indicated significance, the treetop at the $i^{th}$ scale was selected. Otherwise, the treetop at the upper scale ($i+1{)}^{th}$ was retained.

#### 2.2.2. Segmentation of Crowns

##### Overview

The identified treetops were used as seeds in a modified SRG algorithm to produce the final segments of the ITCs. The traditional SRG method was improved in the following ways: (1) a contextual merging criterion was proposed based on neutrosophic logic and (2) a constraint based on the shape of the crown was imposed during the merging process. Starting from the identified treetops, the procedures were carried out iteratively until all of the pixels were merged into a region with a treetop. As described in Section 2.2.1, the identified treetops were regions. At each iteration and for a given treetop region, all of its connected pixels were first evaluated based on the merging criterion defined based on neutrosophic logic, and for those pixels satisfied the merging criterion, an additional condition were checked. The additional condition was based on the assumption that the shape of a crown could be described by a Gaussian function. As a result, these candidate pixels for merging were examined to determine whether or not the crown shape by adding them conformed to the Gaussian template described in Equation (1). The morphology of tree crowns has been extensively examined in template-based ITC delineation studies [11,12] and Gaussian functions have been a popular choice for templates as they are not only fast to compute but also adaptable to the morphologies of different tree species.

##### The Contextual Merging Criterion based on Neutrosophic Logic

As mentioned previously, neutrosphc logic was first used during image segmentation by Shan et al. [25] to extract lesions from noisy ultrasound images. It mitigated the effect of noise on the segmentation results by treating noisy pixels differently. The level of indeterminacy, defined as variance in the neighbourhood of a given pixel, was used to determine whether the pixel was in a noisy area. If it was in a noisy area, the properties of both the pixel and its neighbourhood were considered in a measure of the degree of truth, which determined the similarity between the properties of this pixel and its neighbouring segment. It was assumed that, for a pixel within a noisy area (i.e., with a high level of indeterminacy), there might be a large degree of uncertainty about whether to merge this pixel with its neighbouring segment based on only the properties of this pixel. It was proposed that this decision should instead be made based on the properties of the neighbourhood of the pixel. In the context of ITC delineation, a lower level of indeterminacy implied that the region surrounding the pixel was homogeneous and the pixel was likely located within a crown, while a higher level of indeterminacy indicated that the pixel was very likely located within the boundaries between tree crowns or between a tree crown and the background.

This study developed a rule-based approach based on neutrosophic logic to determine whether a pixel could be merged with its neighbouring segment. Specifically, if the degree of indeterminacy was smaller than the threshold value, the degree of truth of the individual pixel was employed, and otherwise, the degree of the truth of a small region (specifically, a 3 × 3 window) surrounding the pixel was employed instead. For a given pixel positioned at (x,y), the degree of indeterminacy, I(x,y), was defined using Equation (5). The term in parentheses in Equation (5) was proposed by Shan et al. [24] as:

(5) $$I\;\left(x,y\right)=\frac{1}{n}\sum_{i=1}^{n}\left(1-e^{-\frac{\sigma_{i}^{2}}{100}}\right),$$

where n is the number of channels and $\sigma_{i}^{2}$ is the variance for channel i calculated within a 3 × 3 window around a pixel. If the degree of indeterminacy was below 0.5, the degree of truth of the individual pixel was calculated. Otherwise, the degree of truth of the small region around the pixel was used. The degree of truth that a given pixel (x,y), could be merged with segment C based on the features of channel i  was defined as follows [25]:

(6) $$T_{i}\left(x,y\right)=1-\frac{\left|\mu {\left(C\right)}_{i}\;-\rho {\left(x,y\right)}_{i}\right|}{\rho {\left(x,y\right)}_{i}},$$

where $\mu {\left(C\right)}_{i}$ and $\rho {\left(x,y\right)}_{i}$ are the mean intensity of segment C and the intensity value of pixel (x,y) in channel i, respectively. The degree of truth that a neighbourhood (3 × 3 window) around pixel (x,y) could be merged with segment C based on the features of channel i was defined as follows [25]:

(7) $$T_{i}\left(x,y\right)=1-\frac{\left|\mu {\left(C\right)}_{i}\;-\mu {\left(x,y\right)}_{i}\right|}{\mu {\left(x,y\right)}_{i}},$$

where μ(x,y) is calculated within the 3 × 3 window around pixel (x,y). Based on the degree of truth in all channels, a weighted mean was calculated to serve as the final degree of truth, S(x,y):

(8) $$S\left(x,y\right)=\frac{\sum_{i=1}^{n}\frac{T_{i}\left(x,y\right)}{\sigma_{i}^{2}}}{\sum_{i=1}^{n}\frac{1}{\sigma_{i}^{2}}},$$

where $\sigma_{i}^{2}\;$ is the variance in channel i calculated within the 3 × 3 window around pixel (x,y).

Shan et al. [25] proposed that if the degree of truth calculated for pixel (x,y) based on either Equation (6) or Equation (7) was greater than 0.6, the pixel could be merged with the growing region (segment C). The current study found that this threshold value worked for degrees of indeterminacy of less than 0.5, which indicate that the pixel (x,y) was located in a homogeneous area. However, for cases with a large degree of indeterminacy, it was found that the optimal threshold value depended on the degree of indeterminacy. As a result, a thresholding sigmoid function was constructed:

(9) $$F_{sigmoid}={\left(\frac{A_{1}*I\left(x,y\right)}{1+e^{-A_{2}\left(I\left(x,y\right)+A_{3}\right)}}\right)}^{1-I\left(x,y\right)}.$$

The values of $A_{1}$ and $A_{2}$ control the scale and steepness of the sigmoid curve. The value of $A_{3}$ is used to adjust the inflection point at which the curvature of the curve changes. In this study, the values of $A_{1}$, $A_{2}$, and $A_{3}$ were set to 1, 1, and 4.5, respectively. If S(x,y) exceeded the threshold value calculated by Equation (9), the pixel was merged with the nearby segment.

##### Constraint based on Crown Shape

**Figure 7:**
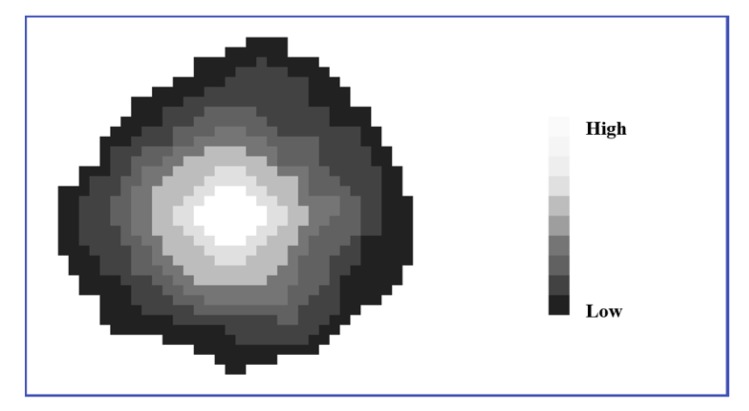
The contour of a typical tree crown in the CHM. The contour lines illustrate points (pixels) of constant value in the CHM. The most inner contour represents the highest points just around the treetop and the outermost contour represents the lowest points around the boundary of the crown.

During the region growing process based on the previously described criteria, the shape information of crowns in the CHM was used to preserve the refined boundaries of crowns and prevent over-segmentation in dense tree clusters. As shown in Figure 7, points neighbouring an initially identified treetop reflect a general decreasing trend in height until the boundary of the crown is reached. This shape constraint was found to be most applicable to CHM-based ITC delineation approaches as the subsequent returns that could represent inconsistent elevation trends within a single crown, were removed. Furthermore, the effects of scan lines and noise in the CHM were removed by using a smoothing process.

During SRG, the immediate neighbours of the growing regions (i.e., pixels along the contours of the regions) were stored as pixels of interest. Then, they were examined to determine whether they conformed to the Gaussian template computed in the treetop detection module described in Equation (1). As noted earlier, the morphology of tree crowns has been extensively examined in template-based ITC delineation studies and Gaussian functions have been a popular choice for templates as they are not only fast to compute but also adaptable to the morphologies of different tree species. However, re-computation of the template after each pixel insertion was expensive. Hence, instead of re-fitting the template each time, the difference in tree height between immediate neighbours and the contour of the region was computed using the CHM. The difference in height was then checked against a threshold buffer zone. Immediate neighbours were considered valid if the absolute difference between the pixel and outermost contour met a given threshold. In the case of tree clusters, any sudden change in height would indicate the presence of another crown type and hence the pixel would not be considered a valid neighbour for the growing region.

### 2.3. Accuracy Assessment

**Figure 8:**
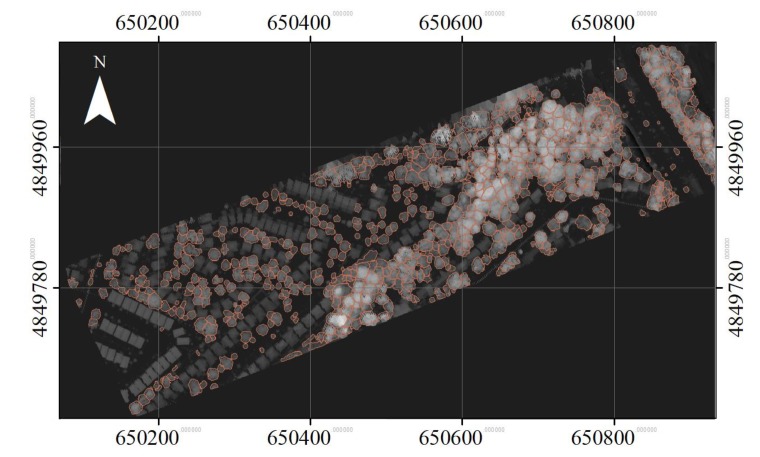
Manually delineated ITCs overlaid on the CHM. The grey level from black to white indicates the elevation from low to high elevation, respectively.

To evaluate quantitatively the segment results, manual delineation was independently carried out on the LiDAR data. A total of 718 trees (hereafter referred to as “reference crowns”) were identified, as shown in Figure 8. The overlap of each reference crown with individual segments was calculated based on Equation (10). It was assigned to one of the following categories [12,32,33,34]: (1) matched, if its overlap with a segment was over 50%; (2) partially matched, if the overlap was less than 50% but greater than 25%; and (3) omitted, if the overlap with any segment was less than 25%. Overlap was determined as follows:

(10) $$Overlap=100\%*\frac{Area\left(Segment\cap^{\;}Reference\;crown\right)}{Area\left(Reference\;crown\right)}$$

In addition, to evaluate the effectiveness of the shape constraint posed while delineating crown boundaries during SRG, we implemented a variant of the proposed method in which the shape constraint was removed. For simplicity and clarification, the proposed method is referred to as “the neutrosophic method with intensities and CHM” and the variation is referred to as “the neutrosophic method with intensities only.” Furthermore, we assessed a variant in which the CHM was used for segmentation with neutrosophic logic instead of intensities. This variant is referred to as “the neutrosophic method with CHM only.” The results obtained from the proposed method and its variants were compared with the MCW segmentation. MCW segmentation is commonly performed in ITC delineation, and many studies have reported that it produces high accuracy, particularly for LiDAR data [10,11]. The MCW method was tested using intensities and CHM individually and combined. The methods are referred to as “MCW with intensities and CHM,” “MCW with intensities only,” and “MCW with CHM only.”

## 3. Results and Analysis

### 3.1. Detected Tree Tops

**Figure 9:**
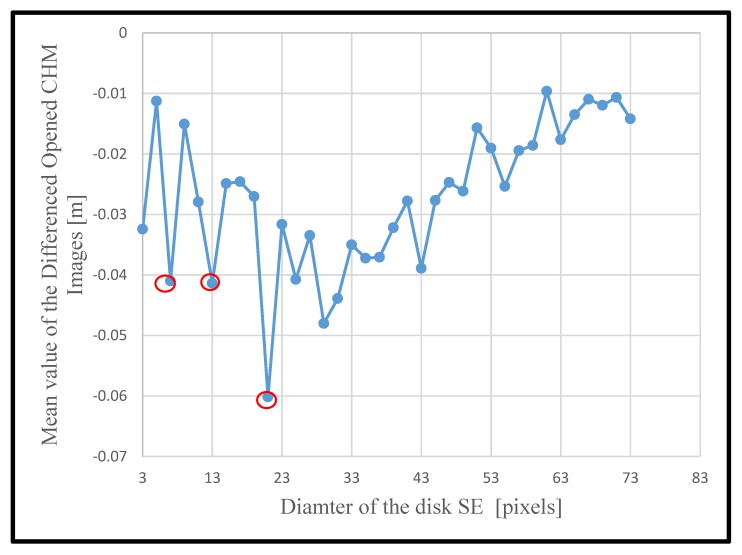
The mean values of the differenced opened CHM images at multiple scales. The scale (i.e., the diameter of the SE) was incremented with a gap of 2 pixels with a starting scale of 3 pixels and final scale of 73 pixels. The local minima, corresponding to the predominant crown sizes, are indicated by red circles. The final scales identified from the mean value analysis were 7, 13 and 21.

To determine the dominant crown sizes in the scene, the morphological operations described earlier were applied to the CHM for channel 1. The results are shown in Figure 9. Several minima were observed, and those corresponding to the predominant crown sizes are marked with red circles. The first minimum, which corresponded to the diameter of the SE of three pixels, was interpreted as upper tree branches and thus was excluded from the analysis. Local minima beyond the diameter of 21 pixels were subdued and hence were interpreted as tree clusters in the scene. Scale sizes of 7, 13, and 21 pixels were identified as representing the predominant small, medium, and large crown sizes in the scene. The effect of the selection of the range of the scale sizes on the results is further discussed in the discussion section. The identified scales were used to generate morphologically opened CHM images, and local maxima were identified as treetops in the opened images.

**Figure 10:**
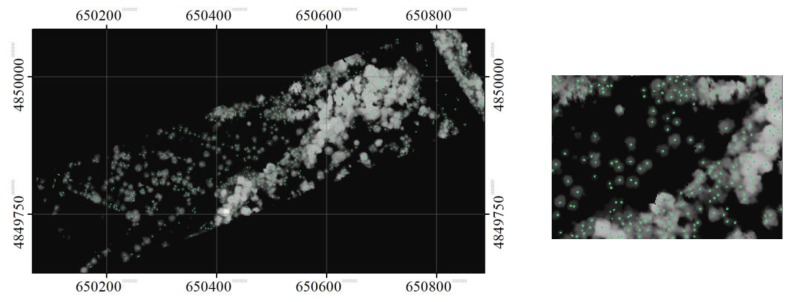
The identified local maxima (treetops), overlaid on the masked CHM, in the opened image with a disk SE with diameter of 7 pixels. A zoomed area is also shown here.

**Figure 11:**
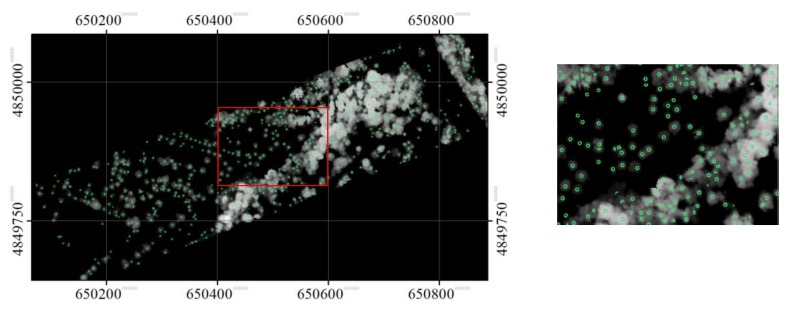
The identified local maxima (treetops), overlaid on the masked CHM, in the opened image with a disk SE with diameter of 13 pixels. A zoomed area is also shown here.

**Figure 12:**
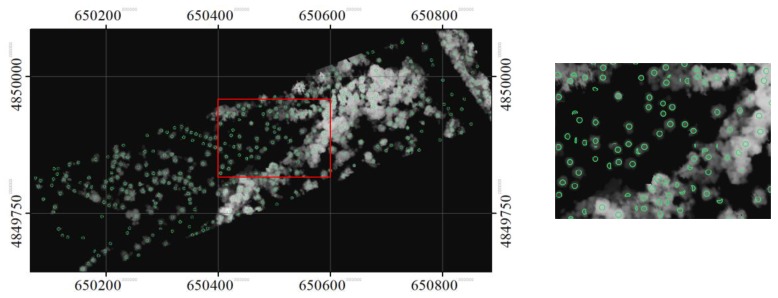
The identified local maxima (treetops), overlaid on the masked CHM, in the opened image with a disk SE with diameter of 21 pixels. A zoomed area is also shown here.

The treetops identified at the three scale sizes are shown in Figure 10, Figure 11, and Figure 12. As expected, some tree crowns appeared only at the small crown scale. For some tree crowns, they appeared in two or three scale sizes. In the latter, the disk SE with the smallest diameter sliced the top part of the tree crown, while the SE with a larger diameter sliced the same crown at a lower height. For some crowns, the SEs at the smaller scale size sliced only parts of the crowns, while the SEs at a larger scale size sliced a tree cluster.

**Figure 13:**
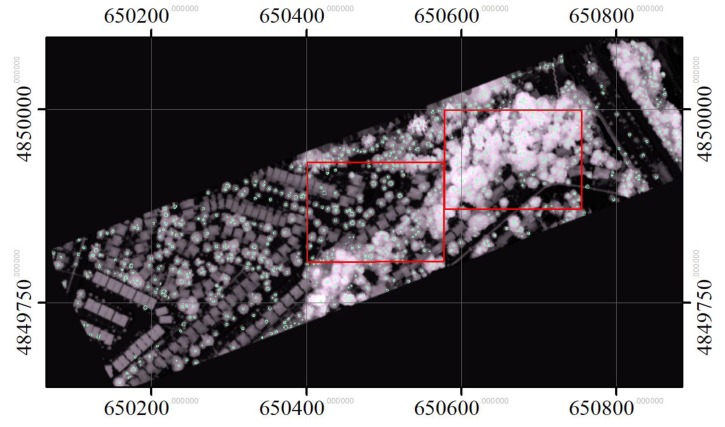
The treetops identified by the proposed multi-scale treetop detection method for the whole study area and two zoomed-in areas representing typical street and woodlot trees.

**Figure 14:**
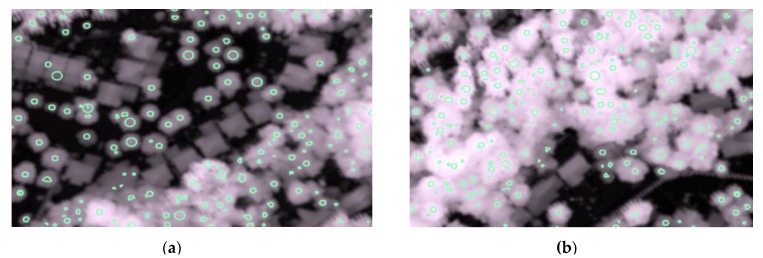
The treetops identified by the proposed multi-scale treetop detection method for the two zoomed areas (shown in Figure 13) representing typical street (**a**) and woodlot (**b**) trees.

The proposed merging strategy consolidated the treetops detected at these three scales in order to retain only unique tree crowns. The merged treetops are shown in Figure 13. Two areas representing typical street and woodlot trees are shown in Figure 14. As expected, the results for trees along streets were almost entirely accurate, and the results for the woodlot areas were reasonably good.

### 3.2. Delineated Individual Tree Crowns

**Figure 15:**
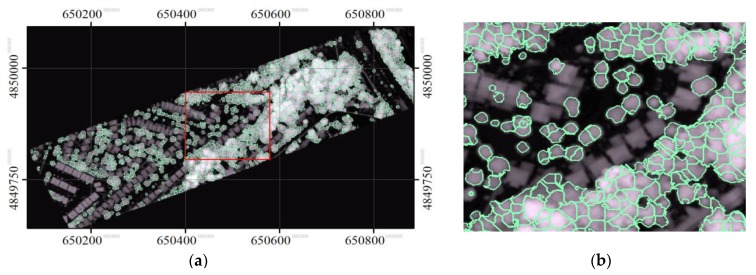
The delineated segments using the proposed method (“the neutrosophic method with intensities and CHM”). The intensity data were used to perform the segmentation using the neutrosophic logic and the CHM was used to enforce the LiDAR shape constraint. (**a**) the whole study area and (**b**) a focused area.

**Figure 16:**
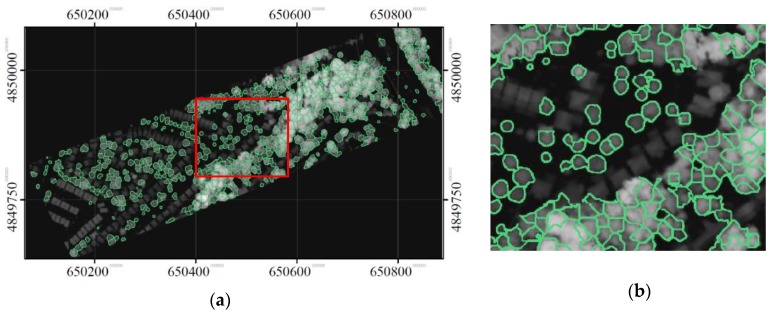
The delineated segments using “the neutrosophic method with CHM only”: (**a**) the whole study area and (**b**) a focused area.

**Figure 17:**
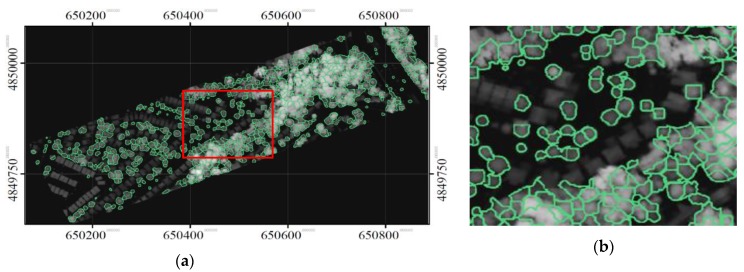
The delineated segments using “the neutrosophic method with intensities only”: (**a**) the whole study area and (**b**) a focused area.

Using detected treetops as seeds, the final delineated tree crowns were identified using the neutrosophic method with intensities and CHM, and are shown in Figure 15. Figure 16 and Figure 17 show the results generated by “the neutrosophic method with CHM” only and “the neutrosophic method with intensities” only, respectively, when the same treetops were used as seeds. As expected, all the methods delineated the crowns of street trees well, and the neutrosophic method with intensities and CHM performed better than its two variants. Table 1 shows the results of the accuracy assessment that compared the segments delineated by the proposed method (“the neutrosophic method with intensities and CHM”) with manually delineated segments (considered as reference). When matched and partially matched crowns were considered together, the producer’s accuracy was 80% and 58% of crowns were matched. The user’s accuracies were 84 and 93% considering only matched and partially matched crowns together, respectively. The user’s accuracies were higher than the producer’s accuracies. In comparison, the accuracies in the segmented results produced by the proposed method and its two variants, and MCM segmentation methods are shown in Table 2. For this comparison, only producer’s accuracy was used. For simplicity, hereafter, unless specified, accuracy refers only to producer’s accuracy. The accuracy of “the neutrosophic method with CHM” only was 72%, and 57% and 15% of the crowns were matched and partially matched, respectively. The accuracy of “the neutrosophic method with intensities” only was 69%, and 56% and 12% of the crowns were matched and partially matched, respectively. These results indicate that neutrosophic logic was able to yield segmentation results by incorporating a measure of contextual information in the merging criterion during region growing. The neutrosophic-logic-based merging criterion was also demonstrated to work on CHM, but incorporating the LiDAR shape constraint was demonstrated to be a better metric for exploiting positional information in the CHM.

Two scenarios led to low accuracy in crown matching and, consequently, a higher number of complete omissions. The first scenario, though not as common in this study, was complete omission of treetops during the treetop identification phase. If a treetop was falsely omitted (i.e., the crown was not localized), the crown was never segmented. The second, and more relevant, scenario was one in which a large commission error arose during the treetop detection phase. False identification of multiple treetops as a single crown led multiple segments to intersect a common reference polygon and none of the segments to be a match or partial match. This was more prevalent in dense forest stands, as multiple treetops were falsely identified due to the complex branch structure of overlapping tree crowns. The relative contribution of multi-scale treetop detection and region growing crown delineation are discussed later in detail.

Table 2 clearly shows that the accuracies of the three MCW methods were relatively low. The highest accuracy was obtained by the MCW method using the CHM only (0.70 for matched and partially matched crowns). However, the neutrosophic method with intensities and CHM achieved 13% more matched crowns. Even though the proposed method and its variants generally outperformed the MCW methods, the results of the three SRG variants indicated that intensity information alone was not sufficient for obtaining accurate ITC delineation results. In both the neutrosophic method with intensities only and the MCW method with intensities only, intensity information produced the lowest accuracy metrics and the highest number of “omitted crowns”. In contrast, the variants using CHM yielded higher accuracy with fewer “omitted crowns”. For the neutrosophic method, the difference between the intensity (a total of 404 matched crowns) and CHM (a total of 407 matched crowns) variants was not statistically significant due to the incorporation of contextual information in the neutrosophic logic-based merge criterion during region growing. The missing contextual information in the MCW segmentations might lead to poor results. To verify the accuracy of the proposed neutrosophic-logic-based region growing method, reference treetops were generated independently of multi-scale treetops by computing the centroids of the reference polygons. The results are shown in Table 3. 

The centroids of the reference polygons were used as reference treetops with a radius of 5 pixels. A total of 718 reference treetops were generated from the reference polygon. “The neutrosophic method with intensities and CHM” led to matching of 88% of crowns. In comparison, 87% and 85% of the 718 crowns were matched by “the neutrosophic method with CHM only” and “the neutrosophic method with intensities only”, respectively. The results revealed a significant improvement with the reference treetops, and fewer cases of over- and under-segmented crowns were present. In comparison with MCW segmentation with intensities and CHM, the proposed method had a 4.2% improvement in matched crowns, although the number of complete omissions remained the same.

## 4. Discussions

Comparing the accuracies reported in Table 1 and Table 2, it is clear that the treetops that were used as seeds and marks in the region growing and MCW segmentation had a significant impact on delineation of ITCs. Even though the multi-scale analysis proposed by Jing et al. (2012) [11] and Hu et al. [12] achieved satisfactory results, it was pointed out that the selection of the scale sizes was critical. Based on our experience with the analysis for the selection of the scale sizes gained through this study and previous studies [11,12], the morphological analysis was able to identify the dominant sizes of objects in a scene. In the context of ITC delineation, the objects could be branches, crowns, or clusters of crowns. As a result, it was important to determine the smallest dominant crown size and the largest crown sizes. In this study, we implemented a new merging criterion based on crown shapes and a statistical test to minimize the effect of the selection of the range of the crown sizes. To investigate the effect of the selected scale sizes on the delineation result, we compared the results using the following scale sizes: (1) 7, 13 and 21 that were proposed in this study; (2) 7, 13 and 29; and (3) 7, 13, 21, and 29. The results are shown in Table 4. Given that the smallest crown size corresponded to a scale size of 7, the results were not sensitive to the selection of the largest scale sizes. 

By implementing a statistical test to determine the suitable scale sizes during the process of merging tree tops, we eliminated the need to select the pre-determined thresholds, similarly to most of the existing methods [11,12,35,36]. In addition, instead of using simple measures such as area and circularities [11,12,35], we employed shapes of crowns by fitting the heights enclosed by detected treetops (cross-sections) to a Gaussian function. This method could be easily extended to use 3D data clouds in the place of CHM. Vega et al. [36] developed a multi-scale method directly applied to a 3D data cloud. Even though satisfactory results were acquired, the selection of the scale, in the form of the number of points, was not trivial and had to be determined by trial and error. Working with 3D data cloud, detailed structural information of tree crowns could be used. However, this required more computation power working with a huge number of data points than with images, and it was challenging to extract useful crown features from the LiDAR returns generated by various objects in a vegetated scene. As a result, for this study, we decided to use the CHM to explore the application of multi-wavelength LiDAR data in ITC delineation. Our results demonstrated the improvement in the ITC delineation by incorporating the intensities and CHM. It is worth mentioning that the contribution of the LiDAR intensities depended on how differences in the radiometric responses among tree crowns [19]. 

With a similar data set (acquired by the same sensor, but over a different area), Dai et al. [19] applied the mean shift segmentation method to delineate individual crowns directly from 3D point clouds, and a support vector machine (SVM) method was used to classify under-segmented areas using LiDAR intensities. The authors [19] pointed out that the reason for using the mean shift method in [19] instead of MCW and region growing methods was the need to know the treetops used as seed points and the difficulty in determinating the treetops. However, for the mean shift method, the horizontal and vertical bandwidth needs to be determined. This is commonly determined based on trial and error. In addition, it may change from one cover type to another. In [19], different sets of values were given to “simple” and “complex” plots, which might cause problems in applying the method to a big area containing mixed “simple” and “complex” plots. In addition, due to the use of the SVM classification, training data were needed. Nevertheless, our results are consistent with those obtained by [19] in terms of the improvement of the ITC delineation by using multi-spectral LiDAR. Due to the difference in the study area, it is hard to perform direct comparisons in terms of segmentation accuracies. In the future work, we will carry out more validation for the proposed method. Neither this study nor Dai et al. [19] employ the colored data clouds (with intensities associated with each LiDAR point) directly. In the future work, we plan to make better use of this. 

## 5. Conclusions

In this study, a framework was proposed to integrate spectral and height information for ITC delineation using multi-spectral LiDAR data. A multi-scale treetop detection method was used to identify crowns with multiple sizes and integrate different scale spaces using Gaussian fitting and a statistical test was implemented in place of pre-determined thresholds. An advanced region-based segmentation method was developed for the segmentation of individual crowns using the detected treetops as seeds. Compared with commonly used region-growing methods, two modifications were proposed to incorporate LiDAR positional and intensity data. First, neutrosophic logic was employed to consider the contextual information and incorporate different spectral channels to derive a combined similarity metric. By incorporating contextual information, the quality of the segmentation was improved. Secondly, a shape constraint was used to prevent over-segmentation of regions when examining the elevation difference between adjacent points. The results were validated using manually delineated reference polygons. A higher number of tree crowns were successfully delineated using the proposed method compared with more commonly used marker-controlled watershed segmentations, and compared with the variants of the proposed methods that only used either CHM or the intensities. The proposed methods were validated with data acquired by a multispectral LiDAR sensor over a study area in the city of Toronto, Ontario, Canada. Compared with the proposed method using either CHM only or using intensities only, the integration of intensities and CHM improved the delineation results significantly (from ~0.7 to 0.8 in accuracy). However, with the commonly used MCM method, the combination of intensities and CHM generated a slightly worse result (with an accuracy of 0.68), compared with that obtained by using the CHM only (0.70). 

The results also showed that with the proposed method, the major factor contributing to the relatively low accuracies considering the “matched crowns” only was the identification of treetops. By using “reference” treetops derived from the manual delineation, the accuracies were improved significantly (from ~0.6 to 0.8). In this study, only the CHM was utilized during the treetop detection phase. Errors might occur due to the complex upper branch structure in dense deciduous crowns that were mis-interpreted as individual trees. In future efforts, LiDAR intensities data can be incorporated to improve the accuracy of the detection of treetops. In addition, as mentioned earlier, we will carry out more validations to the proposed method. 

Even though the proposed method was demonstrated to combine LiDAR height and spectral information with good effect, improvements can still be made to reduce erroneous segmentations and refine treetop identification. In this study, the LiDAR 3D data points (elevation and intensities) were converted to a 2D raster image. In the future work, the 3D information could be used to improve the ITC delineation in two ways: (1) it could be used in post-processing to revise problematic segments by either splitting them or merging them [12], and methods could be developed to use 3D data clouds directly, such as in [19]. In addition, in this study, no a prior knowledge was used. In future work, additional information about the tree crowns or the scenes could be used to determine the dominant crown sizes [37] and information on data quality could be used to improve the determination of the of indeterminacy in the neutrosophic logic.

In future work, the proposed method can be applied to a combined dataset with a CHM derived from LiDAR data and multispectral/hyperspectral data obtained from passive optical imagery. Even though issues regarding co-registration of the two datasets may arise, as outlined in this study, multispectral/hyperspectral can offer richer spectral information, compared with LiDAR intensities.

## Figures and Tables

**Table 1:** Accuracy metrics for ITC delineation using the proposed method based on the reference data.

	Matched	Partially Matched	Omission	Producer’s Accuracy ^1^	Producer’s Accuracy ^2^	User’s Accuracy ^1^	User’s Accuracy ^2^
Reference	419	155	144	0.58	0.80	-	-
Segmentation	487	55	50	-	-	0.84	0.93

^1^ Considering the matched crowns only. ^2^ Considering the matched and partially matched crowns.

**Table 2:** Comparison in the accuracies of the delineated crowns obtained by the proposed method, its two variants, and MCW segmentation, based on the manually delineated crowns.

Method	Matched	Partially Matched	Omitted	Producer’s Accuracy ^1^	Producer’s Accuracy ^2^
Neutrosophic method with intensities & CHM	419	155	144	0.58	0.80
Neutrosophic method with CHM only	407	109	202	0.57	0.72
Neutrosophic method with intensities only	404	89	225	0.56	0.69
MCW with CHM only	323	177	218	0.45	0.70
MCW with intensities only	269	143	306	0.37	0.57
MCW with CHM and intensities	327	164	227	0.46	0.68

^1^ Considering the matched crowns only. ^2^ Considering the matched and partially matched crowns.

**Table 3:** The accuracies of the delineated crowns generated by using reference treetops, as the comparison with those obtained by using automatically detection treetops (Table 2).

Method	Matched	Partially Matched	Omitted	Producer’s Accuracy ^1^	Producer’s Accuracy ^2^
Neutrosophic method with intensities & CHM	635	16	67	0.88	0.91
Neutrosophic method with CHM only	628	14	76	0.87	0.89
Neutrosophic method with intensities only	608	17	93	0.85	0.87
MCW with CHM only	596	54	68	0.83	0.91
MCW with intensities only	604	46	68	0.84	0.91
MCW with CHM and intensities	605	46	67	0.84	0.91

^1^ Considering the matched crowns only. ^2^ Considering the matched and partially matched crowns.

**Table 4:** The accuracies of the delineated crowns generated the proposed method with different scale sizes.

Scale Sizes	Matched	Partially Matched	Omitted	Producer’s Accuracy ^1^	Producer’s Accuracy ^2^
7, 13, 21	419	155	144	0.58	0.80
7, 13, 29	415	142	155	0.58	0.78
7, 13, 21, 29	421	143	160	0.59	0.78

^1^ Considering the matched crowns only. ^2^ Considering the matched and partially matched crowns.
